# A hidden reservoir of antibiotic resistance genes: transferable plasmids in community air and wastewater

**DOI:** 10.3389/fmicb.2026.1699056

**Published:** 2026-03-05

**Authors:** Xinru Luo, Jianrong Hou, Dan Xia, Yong Zhou, Na Huang, Junhua Liu, Xinqiang Zhang, Xia Tao, Anna Wang, Juntao Li, Pengzhe Qin, Xinwei Wu, Peng He

**Affiliations:** 1 School of Public Health, Sun Yat-sen University, Guangzhou, Guangdong, China; 2 Guangzhou Center for Disease Control and Prevention (Guangzhou Health Supervision Institute), Guangzhou, China; 3 Institute of Public Health, Guangzhou Medical University and Guangzhou Center for Disease Control and Prevention, Guangzhou, Guangdong, China

**Keywords:** antibiotic resistance gene, community, drug resistance, particulate matter, transferable antibiotic resistance plasmids

## Abstract

Plasmid-mediated dissemination of antibiotic resistance genes (ARGs) poses a major public health threat. In contrast to the well-studied resistance plasmids within pathogens, those from non-pathogenic environmental reservoirs remain underexplored. Here, we characterized transferable multidrug-resistant plasmids captured from community air and wastewater via conjugation assays. Transconjugants obtained from these environmental samples were profiled phenotypically against 17 antibiotics and genetically via short- and long-read sequencing. Conjugative plasmid transfer was successfully captured from 33 (20.6%) of 160 environmental samples, yielding 78 transconjugant isolates and 40 plasmid types. The captured plasmids conferred resistance to 4–18 antibiotics, with near-universal resistance to ampicillin (98.7%) and retained susceptibility to polymyxin B (84.6%). Among 150 ARG instances identified across 19 classes, *aac(3)-IId* was the most prevalent. The dominant plasmids ps15D023_8 (wastewater) and peccDNA113 (airborne) were particularly notable; peccDNA113 carried 4 ARGs, 9 virulence factors (including *fimH* and *AcrB*), and confers resistance to at least 7 antibiotics. Critically, the carbapenemase gene *blaNDM-5* was detected, and peccDNA113 shows homology to clinical plasmids, indicating a high risk of clinical–environmental exchange. These findings highlight community environments as crucial reservoirs for mobile, high-risk resistance plasmids and underscore the urgent need for expanded surveillance beyond clinical settings.

## 1 Introduction

Antimicrobial resistance (AMR) has emerged as a critical global issue in the 21st century, affecting humans, animals, and the environment (Samreen et al., 2021). The widespread dissemination of ARGs not only accelerates the emergence and spread of resistant bacterial strains but also facilitates the horizontal transfer of resistance among different microorganisms, thereby significantly increasing potential risks to public health and environmental safety (Yan et al., 2021). The World Health Organization (WHO) has identified AMR as one of the most significant threats to human health and as an urgent global health concern (Shao et al., 2021).

ARGs disseminate through diverse environmental mediums, such as water and air (Shao et al., 2018; Abdel-Raouf et al., 2012; Zhou et al., 2023). While studies have detected ARGs in urban settings, such as wastewater treatment systems, hospitals, bathrooms, and outdoor air (Zhang et al., 2018; Wang et al., 2022; Su et al., 2018), research on community environments—particularly airborne ARGs—remains strikingly limited (Yang et al., 2024). This gap is concerning given that community-acquired resistant infections are increasingly linked to higher mortality, morbidity, and healthcare costs (Furuya and Lowy, 2006; Felmingham et al., 2002; Cravo Oliveira Hashiguchi et al., 2019). Notably, antibiotic resistant bacteria in community wastewater and air may directly threaten human health (Hassoun-Kheir et al., 2020; Charles et al., 2022; Mao et al., 2019), with airborne transmission posing a uniquely pervasive exposure risk (Huijbers et al., 2015).

The dynamics of ARG spread are driven by both vertical gene transfer (VGT) and horizontal gene transfer (HGT), though HGT is the primary engine of rapid resistance dissemination (Vikesland et al., 2019; Shi et al., 2022). Unlike VGT, HGT enables bacteria to dynamically adapt through mobile genetic elements, with plasmids serving as key vectors (Agarwal et al., 2023). Critically, plasmids can shuttle ARGs not only between pathogens but also from non-pathogenic to pathogenic bacteria, thereby bridging environmental and clinical resistance pools (Metzker, 2010; Lu et al., 2016). While most studies focus on ARGs in pathogenic (Zhou et al., 2018; Poirel et al., 2010), the transfer potential of plasmids from non-pathogenic reservoirs remains understudied—a knowledge gap with direct implications for resistance containment. Traditional methods for plasmid analysis (e.g., PCR, MLST) are limited by low throughput and incomplete genetic resolution (Jain et al., 2016). In contrast, whole-genome sequencing (WGS) enables comprehensive gene identification, high-resolution typing, and mutation analysis through integration with public databases. The recent advent of long-read sequencing, particularly nanopore metagenomics, has further revolutionized this field by enabling the complete reconstruction of plasmids and other mobile genetic elements directly from complex environmental samples, overcoming the limitations of short-read assemblies in resolving repetitive regions and complex genetic contexts (Peng et al., 2025).

To address these research gaps, this study aimed to investigate transferable antibiotic-resistant plasmids in community air and wastewater. Our strategy involved directly isolating antibiotic-resistant plasmids from environmental samples through conjugation assays and characterizing their resistance profiles via antimicrobial susceptibility testing. We then employed a combination of, short- and long-read sequencing technologies to fully resolve the genetic architectures of these plasmids. Subsequent bioinformatics analyses were conducted to elucidate their genetic features and evolutionary relationships. These findings provide valuable insights into the dissemination mechanisms of antibiotic-resistant plasmids within community environments and contribute a theoretical foundation for understanding and mitigating the transmission of antimicrobial resistance.

## 2 Materials and methods

### 2.1 Recipient strain

The recipient strain used in this study is *Escherichia coli CV601*, a widely applied and well-characterized standardized engineered strain in the field, which does not carry any natural plasmids. Multiple core genetic markers are stably integrated into its chromosome, providing a stable and specifically selective genetic background for conjugation experiments. These markers include: a green fluorescent protein (GFP) gene, which serves as a visualization and screening marker, and a kanamycin resistance gene, which functions as a non-transferable “fixed selection marker” for the recipient strain. The resistance gene and the GFP gene are co-integrated as a single copy within the same transposon structure (Christensen et al., 1998). Additionally, a targeted point mutation in the drug resistance-determining region of the chromosomal rpoB gene confers rifampicin resistance to the strain, representing a classic laboratory-induced resistance mechanism. In experiments, rifampicin and kanamycin are used in combination to ensure recipient strain purity and background screening, while the specific capture of transconjugants requires additional selection based on the resistance carried by the target conjugative plasmid. This well-established system makes CV601 a reliable tool for capturing and characterizing mobile genetic elements from environmental samples.

### 2.2 Sampling sites

Community wastewater and air samples were continuously collected for 8 months across nine districts in Guangzhou. While both sample types were collected within all nine districts, the specific collection sites for wastewater and air were not matched one-to-one. The selected sampling sites encompassed major residential communities, entertainment facilities, commercial centers, educational institutions, and critical transportation hubs. All community sampling sites were chosen to be away from point sources known for high antibiotic resistance prevalence such as hospitals, farms, and wastewater treatment plants to minimize interference from these environments.

### 2.3 Sample collection and cultivation

Wastewater samples (50–100 mL) were collected using sterile pipettes or liquid sampling devices prior to disinfection procedures. Air samples were collected at designated spots using an AirPort MD8 portable air sampler (Sartorius AG, Göttingen, Germany), operating for 2 h at each sampling point. The number of air sampling points was determined based on the size of each area. After collection, all samples were immediately transported to the laboratory on ice for processing and cultivation.

The wastewater samples were centrifuged (Centrifuge Model: Eppendorf 5804 R) at room temperature (4,000 × g, 10 min), and the supernatant was discarded. The sediment was dissolved in 45 mL of 10% Tryptic Soy Broth (TSB; Huankai, China) solution. The suspension was then incubated at 28 °Cfor 24 h with constant shaking at 150 rpm using a shaking incubator (ZHICHENG, China).

The optical density at 600 nm (OD_600_) of the bacterial cultures was measured using a NanoPhotometer® N60 (Implen GmbH, Munich, Germany). Cultures were adjusted to a uniform concentration of OD_600_ = 1.40 ± 0.05 using sterile 0.85% saline. Air samples collected on gelatine membrane filters (Sartorius MD8 gelatin filters) were dissolved in 10 mL of sterile 0.85% saline solution. Wastewater sediment samples were resuspended in sterile 0.85% saline. All subsequent processing steps for these prepared samples were identical.

The processed environmental samples served as the donor community for the subsequent conjugation experiments. No further bacterial isolation, purification, or characterization of individual donor strains was performed, as the objective was to assess the overall horizontal gene transfer potential of the native, complex microbial communities present in air and wastewater.

### 2.4 Conjugation transfer experiment

#### 2.4.1 Experimental design and rationale

The objective of this experiment was to capture mobile genetic elements, particularly plasmids, directly from complex environmental microbial communities. Therefore, the processed environmental samples (Section 2.3) were used as the donor community without isolating specific donor bacteria. A plasmid-free, green fluorescent protein (GFP)-labeled *Escherichia coli CV601* strain, with intrinsic resistance only to kanamycin and rifampicin, was used as the recipient to allow for specific selection of transconjugants.

#### 2.4.2 Bacterial culture and quantification

Prior to each mating experiment, the recipient strain *E. coli* CV601 was cultured as described in Section 2.3, and the optical density was adjusted to an OD600 of 1.0 to ensure consistent and reproducible starting conditions. Based on widely validated empirical values for *E. coli*, this corresponds to a cell density of approximately 1 × 10^9^ CFU/mL.

#### 2.4.3 Filter mating procedure

The filter mating conjugation was performed using a two-step incubation protocol (liquid pre-mixing followed by solid-surface mating) to maximize conjugation efficiency. First, a conjugation mixture was prepared by combining 2 mL of the processed environmental sample (donor suspension) with 2 mL of the adjusted recipient cell suspension (giving a 1:1 volume ratio) in a sterile tube. This mixture was incubated at 37 °C for 24 h to allow pre-adaptation and initial cell-to-cell contact.

After the liquid pre-incubation, 400 μL of the mixture was filtered onto a sterile membrane filter (0.22 μm pore size). The filter was then placed on a Luria-Bertani (LB) agar plate supplemented with cycloheximide (100 mg/L) to inhibit fungal growth and incubated at 37 °C for an additional 24 h to facilitate plasmid transfer. Based on the initial recipient cell density, the approximate number of recipient cells on the filter at the start of this solid-phase conjugation was 2 × 10^8^ CFU.

#### 2.4.4 Selection and verification of transconjugants

Following solid-phase conjugation, the bacterial biomass on the filter was resuspended in 10 mL of sterile 0.85% saline. To select for transconjugants, 10 μL aliquots of this suspension were spread onto Mueller-Hinton (MH) agar plates containing a triple-antibiotic combination: (1) kanamycin (50 μg/mL) and rifampicin (50 μg/mL) to select for the recipient *E. coli CV601* background, (2) cycloheximide (100 μg/mL) to prevent eukaryotic contamination, and (3) one of ten additional antibiotics at CLSI-based breakpoint concentrations: tetracycline (16 μg/mL), polymyxin B (4 μg/mL), tigecycline (4 μg/mL), ceftazidime (16 μg/mL), meropenem (4 μg/mL), gentamicin (16 μg/mL), ciprofloxacin (1 μg/mL), levofloxacin (2 μg/mL), chloramphenicol (32 μg/mL), or clarithromycin (32 μg/mL). This third antibiotic selected for the transfer of specific resistance determinants. Consequently, the final number of recipient-derived cells plated on each selective plate was approximately 2 × 10^5^ CFU.

Only colonies that grew on these triple-antibiotic plates and exhibited clear green fluorescence under 365 nm UV light were considered putative transconjugants. This two-step verification (triple-antibiotic resistance plus GFP fluorescence) was crucial to conclusively exclude the growth of donor bacteria, spontaneous resistant mutants of the recipient, or other contaminants.

#### 2.4.5 Critical controls

To ensure the specificity of the conjugation assay and rule out other potential explanations for the observed antibiotic resistance, we conducted critical negative control experiments in parallel. The recipient strain (*Escherichia coli CV601*) was plated alone onto the same triple-antibiotic selective agar, using the same plating volume (10 μL of pure culture) and cell number (2 × 10^5^ CFU) as in the conjugation assay. No colony growth was observed on these spontaneous mutation control plates during the 24-h observation period, confirming that spontaneous mutations conferring resistance to the third (selective) antibiotic were not detected under these experimental conditions.

#### 2.4.6 Confirmation and storage

Fluorescent colonies from triple-Ab plates were purified by re-streaking on fresh MH agar plates containing the same three antibiotics. Pure isolates were preserved in 20% (v/v) glycerol at −80 °C for downstream analysis (Wolters et al., 2014). Given that the donor was an uncharacterized community, classical “conjugation frequency” (transconjugants per donor cell) could not be calculated. Results are therefore reported as the number and proportion of environmental samples yielding transconjugants, and the number of transconjugant isolates obtained for each antibiotic selection.

### 2.5 Antibiotic resistance testing

Antimicrobial Susceptibility Testing (AST) was performed according to the guidelines of the Clinical and Laboratory Standards Institute (CLSI, M100-S30, 2020) and the European Committee on Antimicrobial Susceptibility Testin*g* (EUCAST v11.0, 2021). The tests were conducted in triplicate to ensure the accuracy and reproducibility of the results.

The panel of antibiotics consisted of the following 17 antibiotics: clarithromycin (CLR), ampicillin (AMP), meropenem (MEM), cefotaxime (CTX), ertapenem (ETP), trimethoprim-sulfamethoxazole (SXT), cephalothin (CEP), ceftazidime (CAZ), polymyxin B (PB), gentamicin (GEN), ciprofloxacin (CIP), tigecycline (TGC), nalidixic acid (NAL), streptomycin (STR), chloramphenicol (CHL), amikacin (AMK), and tetracycline (TET).

For the transconjugant isolates identified in each conjugation experiment, the minimum inhibitory concentration (MIC) was determined. Briefly, bacterial suspensions were inoculated onto Mueller-Hinton agar plates containing serially diluted antibiotics. The MIC was defined as the lowest antibiotic concentration that completely inhibited visible bacterial growth after incubation at 37 °C for 16–20 h.

### 2.6 DNA extraction and gene sequencing

Plasmid DNA was extracted from the obtained transconjugant isolates using the TIANpure Midi Plasmid Kit (TIANGEN BIOTECH, China) following the manufacturer’s instructions. The concentration and purity of the extracted plasmid DNA were assessed using a *Qubit 4.0 Fluorometer* (Thermo Fisher Scientific, United States) with the Qubit dsDNA HS Assay Kit and a NanoDrop™ One Microvolume UV–Vis Spectrophotometer (Thermo Fisher Scientific).

DNA libraries were constructed only for plasmids DNA extracts that met the predefined quality thresholds: an A_260_/A_280_ ratio between 1.8 and 2.0 and a concentration greater than 50 ng/μL.

Short-read sequencing was performed on an MGISEQ-2000 platform (MGI Tech Co., Ltd., Shenzhen, China) using the MGISP-100 library preparation system. Long-read sequencing was performed on a GridION platform (Oxford Nanopore Technologies, Oxford, UK) using the QNome-3841 library preparation kit (QITAN Technology, Tianjin, China). All sequencing was performed in accordance with the respective manufacturers’ protocols.

### 2.7 Bioinformatics and statistical analysis

Short-read sequencing data underwent quality control (QC) and adapter trimming using Trimmomatic (v0.39). *De novo* assembly of quality-filtered reads was performed with SPAdes (v3.15.5). For hybrid assembly integrating short-read and long-read data, Unicycler (v0.5.0) was run in bold mode with default parameters. Coding sequences were prediction from the assembled contigs using Prokka (v1.14.6) (Seemann, 2014).

Core genome single nucleotide polymorphism (SNP)-based phylogenetic trees were constructed with Snippy (v4.6.0) and visualized using the Interactive Tree of Life (iTOL) online (Letunic and Bork, 2021). ARGs were identified using the Resistance Gene Identifier (RGI) tool (v5.2.1) against the Comprehensive Antibiotic Resistance Database (CARD) (Alcock et al., 2023). Virulence genes were annotated with the Virulence Factors Database (VFDB) (Liu et al., 2022).

Plasmid replicon types were identified using the Mob_recon tool (version 3.19) and the PLSDB database^1^ with thresholds of ≥0.99 identity and ≤0.1 *E*-value. Collinearity analysis of key plasmids was performed with genoPlotR package (v0.8.11) in *R* (v4.3.3; R Core Team, 2023). Circular genome maps were generated using *Proksee* (Grant et al., 2023). To definitively attribute resistance genes to the captured plasmids rather than the recipient chromosome, the genome of the recipient *E. coli* CV601 strain was used as a reference for subtraction during bioinformatic analysis.

All statistical analyses were performed using IBM SPSS Statistics (v27.0; IBM Corp., United States). Chi-square tests were used to assess differences in the distribution of resistance phenotypes and virulence genes between groups derived from different environments (*p* < 0.05). Categorical variables are reported as frequencies and percentages. Visualizations were created using multiple tools: Stacked bar and radar charts were generated using (R v4.2.1) with the ggplot2 package and Heatmaps and Sankey diagrams were created using the TuTu Cloud online platform.^2^

## 3 Results

### 3.1 Samples

A total of 160 community environment samples (124 wastewater samples and 36 air samples) were collected from 9 districts in Guangzhou, China, covering diverse community settings (residential areas, commercial centers, and public spaces). HGT of resistance plasmids was successfully captured from 33 of the 160 samples, representing an overall positive sample proportion of 20.63% (33/160). The success proportion was 19.4% (24/124) for wastewater samples and 25.0% (9/36) for air samples. From these positive samples, a total of 78 transconjugant isolates were obtained (60 from wastewater and 18 from air) for downstream analysis.

### 3.2 Plasmid typing

A total of 78 transconjugant isolates underwent short-read sequencing, with 28 representative isolates selected for additional long-read sequencing. Hybrid assembly of the short-read and long-read sequencing data successfully identified 24 plasmid types, comprising 40 plasmids (Table 1). Plasmid annotation was first performed using mob_recon (v.3.15) for the initial identification. For sequences that could not be annotated, PLSDB database (v.2023_11_03_v2) was then used for further annotation.

**Table 1:** Distribution of plasmids of transconjugants (*n* = 40).

Type of samples	Plasmid	Number	Rate (%)
Wastewater	IncFIA/IncFIB/IncFIC	1	2.50%
	IncFIA/IncFIB/IncFIC/IncFII	1	2.50%
	IncFIA/IncFIB/IncFIC/IncX1	1	2.50%
	IncFIA/IncFIB/IncU	1	2.50%
	IncFIA/IncFIC	2	5.00%
	IncFIA/IncFII	1	2.50%
	IncFIA/IncFII/IncQ1	1	2.50%
	IncFIB/rep_cluster_2244	1	2.50%
	IncI-gamma/K1	1	2.50%
	IncI-gamma/K1/IncY	1	2.50%
	IncN	3	7.50%
	peccDNA113	1	12.50%
	ps15D023_8	5	2.50%
	rep_cluster_1760	1	2.50%
	rep_cluster_312	1	2.50%
	rep_cluster_488	1	2.50%
	rep_cluster_867	1	5.00%
	unnamed15	2	2.50%
Air	IncFIA/IncFIC	1	2.50%
	IncFII/IncN	1	2.50%
	IncW	1	12.50%
	peccDNA113	5	7.50%
	ps15D023_8	3	7.50%
	rep_cluster_1760	3	2.50%

Among the 40 plasmids identified, ps15D023_8 was the most prevalent (20.00%, 8/40), followed by peccDNA113 (15.00%, 6/40). Environment-specific distribution analysis showed that ps15D023_8 was predominant in wastewater-derived plasmids (19.23%, 5/26), while peccDNA113 showed the highest prevalence in air-derived plasmids (35.71%, 5/14). The aforementioned plasmid shares a highly similar (over 99%) backbone region with known plasmids in the database (ps15D023_8 and peccDNA113).

### 3.3 Transconjugant antibiotic susceptibility testing

AST was performed against a panel of 17 antibiotics following CLSI guidelines (Supplementary Table S1). The recipient strain *E. coli* CV601 remained susceptible to all tested antibiotics, confirming its suitability as a recipient in conjugation experiments. In contrast, transconjugant isolates displayed significantly expanded antibiotic resistance profiles, demonstrating the successful horizontal transfer of resistance genes from environmental samples. The number of antibiotic resistances per transconjugant isolate ranged from 4 to 16, with isolates WK3 and WT10 exhibiting the broadest spectrum, demonstrating resistance to 16 antibiotics. The complete phenotypic drug resistance profiles of all transconjugants derived from both air and wastewater mating experiments are summarized in Table 2. Analysis of resistance prevalence revealed that ampicillin showed the highest resistance rate (98.72%, 77/78), followed by cefoxitin (96.15%, 75/78) and amikacin (92.31%, 72/78). In contrast, polymyxin B exhibited the lowest resistance rate (15.38%, 12/78). Among the screening antibiotics, cefoxitin demonstrated the highest resistance prevalence, while clarithromycin showed an overall resistance rate of 26.92% (21/78).

**Table 2:** Overall phenotypic antibiotic resistance profile of transconjugants captured from air and wastewater mating experiments (*n* = 78).

Antibiotic	Number of drug resistances	Resistance rates (%)
AMP	77	98.72%
CEP	75	96.15%
AMK	72	92.31%
NAL	64	82.05%
TET	53	67.95%
ETP	39	50.00%
CTX	33	42.31%
CHL	32	41.03%
SXT	31	39.74%
CAZ	31	39.74%
MEM	31	39.74%
TGC	30	38.46%
CIP	30	38.46%
STR	28	35.90%
GEN	27	34.467%
CLR	21	26.92%
PB	12	15.38%

Based on the AST results, we classified 14 antibiotics as high-risk for environmental dissemination due to high resistance rates among transconjugant isolates. These high-risk antibiotics included eight agents associated with airborne transmission and respiratory infections—ampicillin, cephalexin, ceftriaxone, ceftazidime, ertapenem, meropenem, ciprofloxacin, and clarithromycin. Five wastewater-associated antibiotics demonstrated prevalence in aquatic environments and carried transferable resistance genes—sulfamethoxazole/trimethoprim, tetracycline, amikacin, gentamicin, and streptomycin.

Comparative phenotypic analysis of antibiotic susceptibility revealed statistically significant differences (all *p* < 0.05) between air- and wastewater-derived transconjugant isolates for six antibiotics—nalidixic acid, ertapenem, ceftriaxone, ceftazidime, meropenem, and ciprofloxacin (Table 3).

**Table 3:** Comparison of antibiotic resistance in wastewater and air samples.

Antibiotic	Wastewater (resistant/non-resistant)	Air (resistant/non-resistant)	*χ*^2^ value	*p*-value	Significance
AMP	17/1	59/1	0.838	0.360	ns
CEP	18/0	55/5	1.603	0.206	ns
AMK	17/1	53/7	0.562	0.454	ns
NAL	18/0	44/16	6.039	0.014	*
TET	13/5	40/20	0.196	0.658	ns
ETP	4/14	35/25	7.222	0.007	*
CTX	2/16	31/29	9.330	0.002	*
SXT	4/14	27/33	3.000	0.083	ns
CAZ	3/15	28/32	5.204	0.023	*
MEM	1/17	29/31	10.705	0.001	*
CIP	12/6	18/42	7.865	0.005	*
STR	4/14	25/35	2.241	0.134	ns
GEN	5/13	22/38	0.483	0.487	ns
CLR	3/15	18/42	1.251	0.263	ns

Significance: Use “*” to indicate *p* < 0.05 (statistically significant), and “ns” to indicate non-significant results.

These results confirm efficient acquisition of diverse antibiotic resistance determinants through conjugation events and reveal distinct environment-specific dissemination patterns. Environmental samples serve as important reservoirs of transferable resistance genes. Complete resistance profiles appear in Supplementary Table S1 with statistical comparisons in Table 3.

Given the importance of quinolone drugs in clinical and environmental settings, we performed an in-depth genotype–phenotype association analysis on all transconjugants exhibiting NAL resistance (*n* = 14). The results showed that a total of 14 transconjugant isolates demonstrated resistance to NAL, with a *qnrS1* gene carriage rate of 35.7% (5/14). This resistance gene belongs to the plasmid-mediated quinolone resistance (PMQR) gene class. In contrast, the remaining 64.3% (9/14) of isolates primarily carried genes such as *Ecol_AcrR_MULT, Ecol_emrE*, and *AcrB* (Supplementary Table S3).

### 3.4 Detection of ARGs in resistant bacteria

Analysis of 40 plasmids revealed that antibiotic resistance genes (ARGs) were detected on 33 of them, while the remaining seven plasmids carried no identifiable ARGs. Among these 33 plasmids, we identified a total of 45 distinct gene types (Table 4). The *aac(3)-IId* gene type exhibited the highest prevalence, detected in 27.27% of the ARG-positive plasmids (9/33). Other frequently identified gene types included tet(A), Ecol_EFTu_PLV, and TEM-1, each present in 24.24% of the plasmids (8/33). A critical finding was that 12 of the 45 distinct gene types (26.67%) are known to confer resistance to more than one class of antibiotics. The co-localization of several such multidrug-resistant bacteria (MDR, defined as resistance to three or more classes of antibiotics) genes on individual plasmids is illustrated in the genetic map of a representative multi-drug resistance plasmid (Supplementary Figure S1).

**Table 4:** Distrubtion of ARGs (*n* = 33).

Category of ARGs	Number of positive samples (*n* = 33)	Constituent ratio (%)	Drug class
aac(3)-IId	9	27.27%	Aminoglycoside antibiotic
Ecol_EFTu_PLV	8	24.24%	Elfamycin antibiotic
TEM-1	8	24.24%	Monobactam; cephalosporin; penam; penem
tet(A)	8	24.24%	Tetracycline antibiotic
sul2	7	21.21%	Sulfonamide antibiotic
QnrS1	6	21.21%	Fluoroquinolone antibiotic
aadA2	6	18.18%	Aminoglycoside antibiotic
AcrB	6	18.18%	Fluoroquinolone antibiotic; cephalosporin; glycylcycline; penam; tetracycline antibiotic; rifamycin antibiotic; phenicol antibiotic; disinfecting agents and antiseptics
Ecol_AcrA	6	18.18%	Fluoroquinolone antibiotic; cephalosporin; glycylcycline; penam; tetracycline antibiotic; rifamycin antibiotic; phenicol antibiotic; disinfecting agents and antiseptics
Ecol_AcrR_MULT	6	18.18%	Fluoroquinolone antibiotic; cephalosporin; glycylcycline; penam; tetracycline antibiotic; rifamycin antibiotic; phenicol antibiotic; disinfecting agents and antiseptics
Ecol_emrE	6	18.18%	Macrolide antibiotic
dfrA12	5	18.18%	Diaminopyrimidine antibiotic
mphA	5	15.15%	Macrolide antibiotic
Mrx	5	15.15%	Macrolide antibiotic
APH(6)-Id	4	15.15%	Aminoglycoside antibiotic
APH(3″)-Ib	4	12.12%	Aminoglycoside antibiotic
qacEdelta1	4	12.12%	Disinfecting agents and antiseptics
sul1	4	12.12%	Sulfonamide antibiotic
NDM-5	3	9.09%	Carbapenem; cephalosporin; cephamycin; penam
dfrA17	3	9.09%	Diaminopyrimidine antibiotic
cmlA1	3	9.09%	Phenicol antibiotic
sul3	3	9.09%	Sulfonamide antibiotic
aadA	2	9.09%	Aminoglycoside antibiotic
aadA23	2	6.06%	Aminoglycoside antibiotic
APH(3′)-Ia	2	6.06%	Aminoglycoside antibiotic
rmtB	2	6.06%	Aminoglycoside antibiotic
others	23	69.70%	/

### 3.5 Correlation between plasmid types and resistance genes

**Figure 1:**
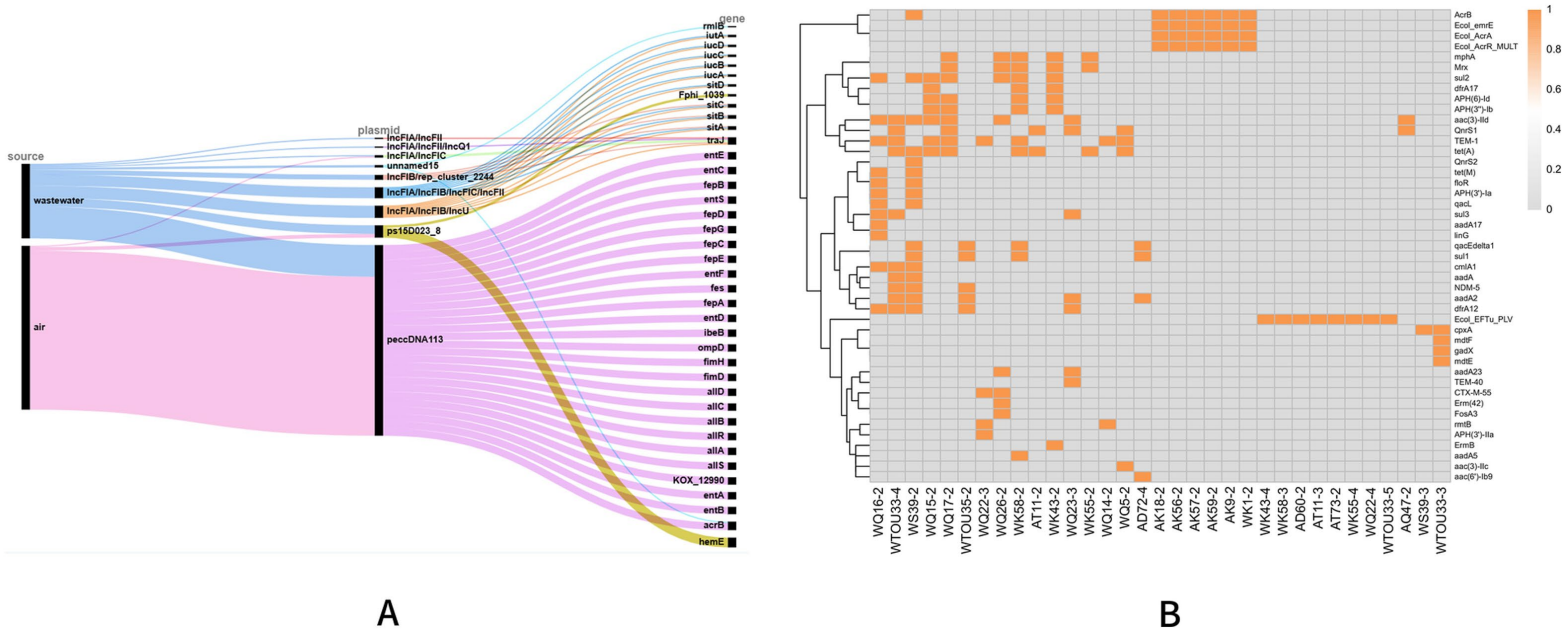
Virulence genes of plasmids and heat map of resistance genes. **(A)** Sankey diagram of virulence genes illustrating the relationships among sample types, plasmid types, and identified virulence genes. The flow proceeds from sample types (left) to plasmid types (middle) and then to virulence genes (right). **(B)** Heat map of resistance genes showing the distribution of ARGs across plasmid variants. Each column represents a specific resistance gene, and each row corresponds to a plasmid variant. Orange indicates the presence of the gene, while gray denotes its absence.

Analysis of plasmid sequences (Figure 1) revealed distinct associations between plasmid types and antibiotic ARGs. Plasmids derived from air samples predominantly clustered with the peccDNA113 plasmid, which harbored multiple ARGs, including *Ecol_emrE*, *AcrB*, *Ecol_acrA*, *Ecol_AcrR_MULT*, and *Ecol_EFTu_PLV*. Functional annotation indicated that these ARGs conferred resistance to 18 antibiotics, spanning cephalosporins, fluoroquinolones, penicillins, tetracyclines, carbapenems, and macrolides.

Comparison of transconjugant isolate phenotypes with their corresponding plasmid ARGs demonstrated strong concordance in most cases (Supplementary Table S2). However, six transconjugant isolates (WK5-2, AD60-2, AT11-3, AT73-2, WK55-4, and WQ22-4) exhibited antibiotic resistance phenotypes that could not be fully explained by the detected plasmid-borne ARGs, suggesting potential contributions from chromosomal mutations, integrative conjugative elements (ICEs) or unannotated resistance mechanisms.

These findings highlight the role of specific plasmid types, such as peccDNA113, in disseminating multidrug resistance while also indicating that transconjugant isolates may acquire resistance through additional genetic determinants beyond plasmid-mediated ARGs.

### 3.6 Plasmid-encoded virulence genes

Analysis of plasmid sequences (Figure 1) revealed that the peccDNA113 plasmid carried the highest number of virulence genes, with up to 26 distinct genes annotated on a single plasmid. This plasmid was detected in transconjugant isolates from both air and wastewater, with an 83.33% (5/6) detection rate in air-derived isolates.

Among the 40 complete plasmids analyzed, 25 contained virulence genes, totaling 74 unique types. The most prevalent virulence genes included *AcrB*, *fimD*, *fimH*, and *hemE,* each had a detection rate of 17.50% (7/40) (Supplementary Table S3). In contrast, 36 virulence gene types were identified at lowe frequencies, each present in only one plasmid.

These findings demonstrate that plasmids serve as important vectors for virulence gene dissemination in environmental bacterial populations, with certain plasmid types like peccDNA113 exhibiting particularly high virulence potential. The complete virulence gene profiles are detailed in Supplementary Table S3.

### 3.7 Alignment results of plasmid peccDNA113

**Figure 2:**
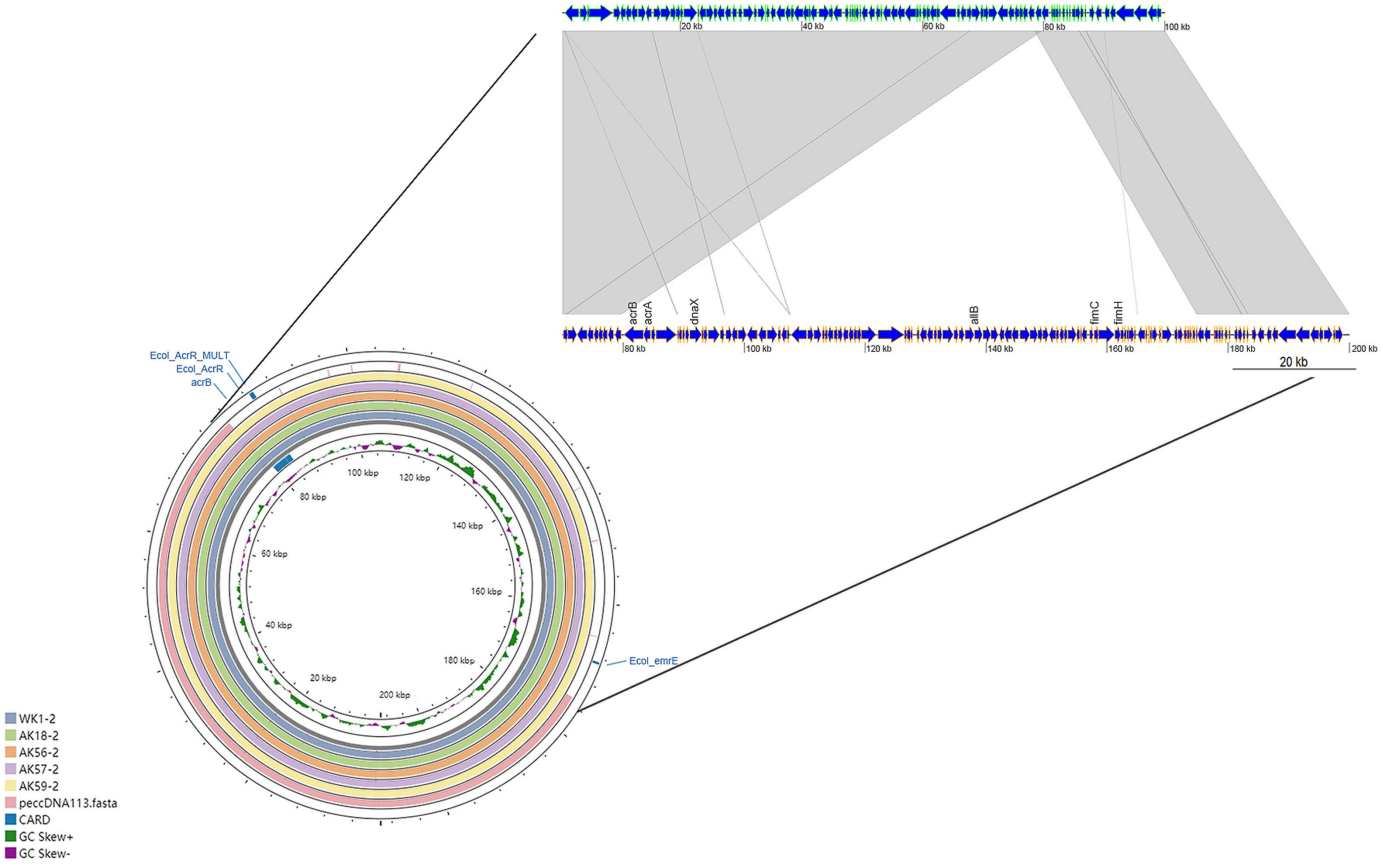
peccDNA113 genome alignment circle diagram and Collinear Comparison between peccDNA113 and AK9-2. Relevant genes are displayed in the outer ring, with ARGs highlighted in blue. The innermost two rings indicate size scale and GC content. The colored ring at the center represents plasmid AK9-2 (this study), while the outer colored rings represent reference plasmids in the following order from inside to outside: WK1-2, AK18-2, AK56-2, AK57-2, and peccDNA113. In the linear alignment, the upper sequence corresponds to plasmid peccDNA113 (GenBank accession no. NZ_CP110860.1), and the lower sequence corresponds to AK9-2. Gray shading indicates aligned regions between the two sequences, while gaps represent insertion sequences.

Given the high prevalence of the peccDNA113 plasmid in community air samples, we performed detailed genomic analysis. Whole-genome alignment revealed six plasmids (AK9-1, AK18-2, AK56-2, AK57-2, AK59-2, and WK1-2) exhibited greater than 99.99% sequence similarity to the peccDNA113 plasmid originally isolated from a pond in California, United States (Figure 2). These highly conserved plasmids consistently carried key virulence and ARGs, including *AcrB*, *Ecol_AcrR*, and *Ecol_AcrR_MULT*.

Linear sequence alignment of a representative peccDNA113 plasmid (AK9-2) against the reference genome (Figure 2) identified an insertion sequence containing functionally important genes such as *DnaX*, *AcrB*, *allB*, *fimC*, and *fimH*.

This conserved genomic architecture suggests the peccDNA113 plasmid serves as a stable vector for disseminating both antibiotic resistance and virulence determinants across geographic regions. The high sequence conservation greater than 99.99% among environmental and clinical plasmids indicates recent global transmission of this mobile genetic element.

### 3.8 Evolutionary analysis

**Figure 3:**
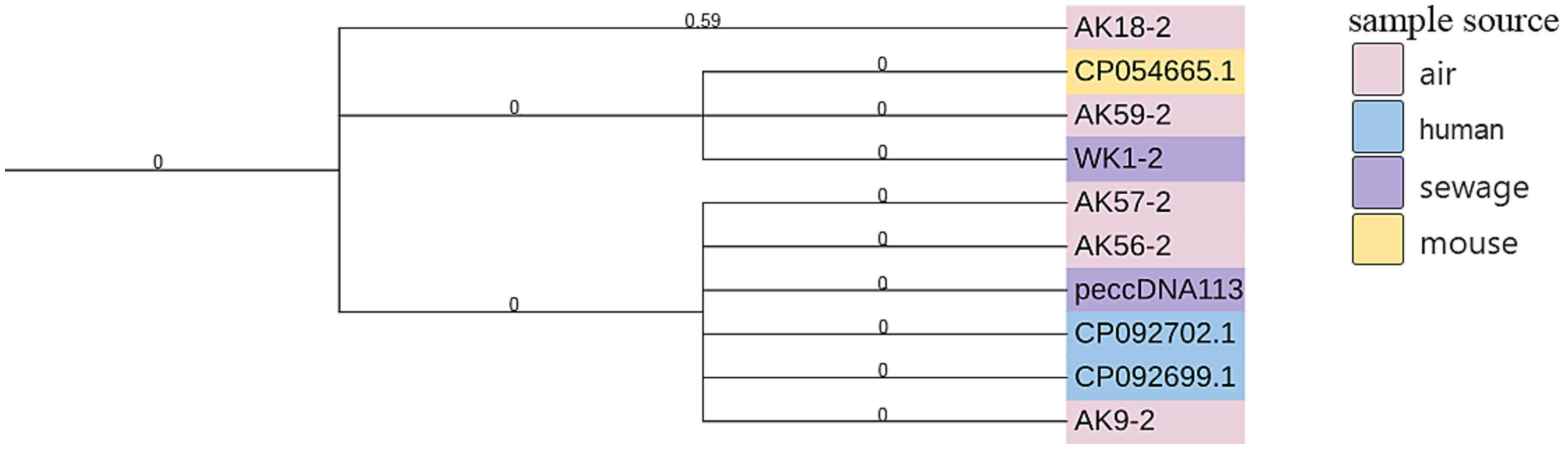
Phylogenetic tree of plasmid peccDNA113. A phylogenetic tree was constructed based on SNP analysis of peccDNA113. Six peccDNA113 plasmids were extracted in this study, while additional sequences were obtained from GenBank (see Supplementary Table S4). SNPs were identified using the Snippy pipeline, with plasmid AK18-2 serving as the reference. Color coding indicates the source of each plasmid: pink for air-derived peccDNA113 strains, purple for wastewater-derived strains, yellow for mouse-derived strains, and blue for *Homo sapiens*-derived strains.

**Figure 4:**
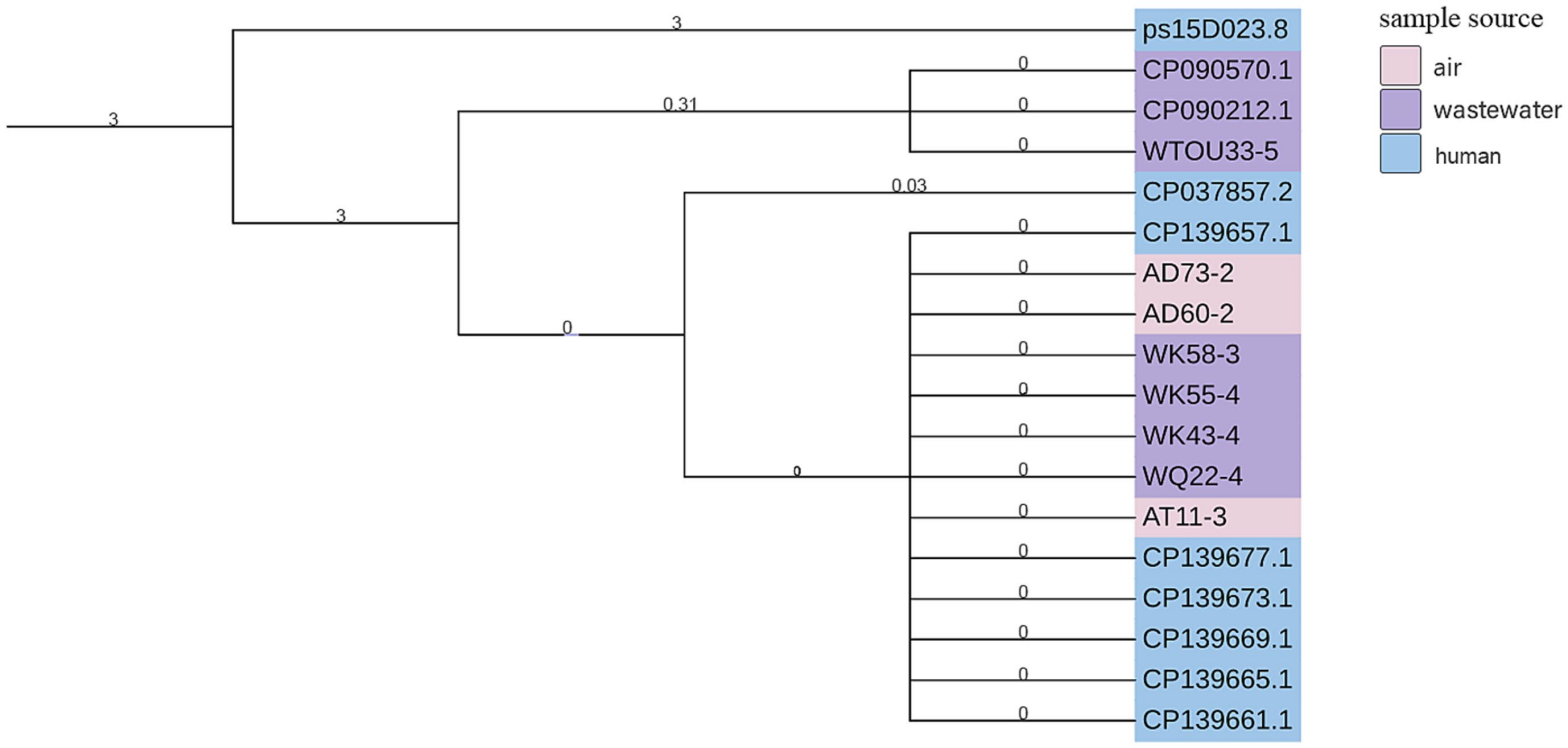
SNP analysis of plasmid ps15D023_8. A phylogenetic tree was constructed based on SNP analysis of ps15D023_8. Eight ps15D023_8 strains were obtained in this study, with additional strains retrieved from GenBank (see Supplementary Table S4). SNPs were identified using the Snippy pipeline, with ps15D023_8 (GenBank accession no. CP101347.1) as the reference. Color coding indicates the source of each strain: pink for air-derived strains, purple for wastewater-derived strains, and blue for *Homo sapiens*-derived strains from hospitals in China.

Phylogenetic analysis of the two most prevalent plasmids (peccDNA113 and ps15D023_8) revealed significant evolutionary relationships between plasmids from community wastewater and air samples (Figures 3, 4).

For peccDNA113, the SNP-based phylogenetic tree demonstrated that plasmid AK59-2 from air samples and WK1-2 from wastewater clustered closely with reference plasmid CP054665.1 from a mouse specimen in Portugal. Additionally, air-derived plasmids AK56-2, AK9-2 and AK57-2 formed a distinct clade with clinical plasmids CP092702.1 and CP092699.1 from human tissue specimens in Sudan.

Analysis of ps15D023_ showed that wastewater-derived plasmid WTOU33-5 clustered with wastewater-origin plasmids CP090570.1 and CP090212.1 from China. Airborne plasmids AD73-2, AD60-2, and AT11-3 shared evolutionary proximity with wastewater plasmids WK55-4, WK43-2, and WQ22-4. All plasmids in this clade originated from Chinese clinical specimens and exhibited high sequence similarity to our experimental isolates.

These phylogenetic relationships demonstrate the cross-environmental transmission and evolution of clinically relevant plasmids, highlighting their role in disseminating antimicrobial resistance across ecological niches and geographic regions.

## 4 Discussion

Multidrug-resistant bacteria (MDR), exhibiting resistance to three or more classes of commonly used antibiotics, contribute to elevated morbidity, therapeutic challenges, and increased mortality (Seemann, 2014); their emergence is strongly associated with the extensive misuse of broad-spectrum antibiotics. While research on MDR within hospital settings is relatively extensive, studies concerning MDR in community environments, particularly involving airborne strains, remain notably limited. Furthermore, since ARGs from non-pathogenic bacteria can be transferred to pathogenic species via HGT, plasmids harboring these transferable resistance genes constitute a primary concern. Consequently, this study characterizes these transferable antibiotic resistance plasmids recovered from the community environment, addressing a critical knowledge gap and highlighting the community environment as a crucial reservoir for emerging MDR threats, distinct from yet interconnected with clinical settings.

Across all environmental samples, plasmid type ps15D023_8 emerged as the predominant lineage, followed by peccDNA113, suggesting both possess significant transmission advantages within the community setting. Stratified by environmental medium, ps15D023_8 demonstrated the highest relative abundance in wastewater samples. This plasmid was initially identified in 2022 within a human-derived Salmonella isolate, a common foodborne pathogen causing enteric infections in humans and animals. Its elevated abundance in wastewater not only reflects substantial environmental adaptability but also implies its potential entry into the human gut microbiota via pathways such as the food chain, facilitating sustained dissemination. Similarly peccDNA113 constituted the predominant type in air samples. Notably, this plasmid (or its core genetic backbone) has been detected in various environmental media such as wastewater and air, and shows the highest detection rate especially in airborne environmental samples, combined with the insidious nature of airborne transmission, significantly elevates the risk of human exposure to resistant genes through inhalation (Huijbers et al., 2015). Current research on airborne resistance plasmids remains limited; thus, our findings contribute insights into their transmission mechanisms. A significant divergence in plasmid types was observed between wastewater and air, wastewater harbored predominantly ps15D023_8, possessing strong clinical associations, while air was dominated by the highly mobilizable peccDNA113. This drastic shift underscores the substantial influence of environment on plasmid ecology. Our comparative approach provides a novel ecological perspective on how selective pressures in different environmental niches drive the evolution and dissemination of distinct plasmid populations. Furthermore, the detection of composite replicons (e.g., IncFIA/IncFIB/IncFIC) highlights the structural complexity of these plasmids, potentially enhancing their environmental fitness (Simner et al., 2023).

AST revealed statistically significant differences (*p* < 0.05) in resistance to nalidixic acid (NAL), ertapenem (ETP), ceftriaxone (CTX), ceftazidime (CAZ), meropenem (MEM), and ciprofloxacin (CIP) between air and wastewater samples. This validates that the environmental matrix drives the selective dissemination of resistance plasmids. Concurrently, the AST results demonstrated near-universal resistance to ampicillin among transconjugants isolated from the community environment, indicating widespread accumulation of resistance resulting from the overuse of β-lactam antibiotics. Notably, polymyxin B retained high efficacy, suggesting its potential as a reserve therapeutic option against MDR pathogens. To further explore the genetic basis of these phenotypic characteristics, we conducted a detailed analysis of the transconjugants. The analysis of nalidixic acid resistance revealed a noteworthy observation: among the resistant transconjugants, the resistance in some strains (5/14) originated from the acquired exogenous gene qnrS1, while the remaining resistant strains (9/14) did not carry any of the targeted plasmid-mediated quinolone resistance genes, including *qnrS1, qnrA, qnrB, qnrC, qnrD, qepA,* and *aac(6′)-Ib-cr*. This may be due to the presence of genes such as *Ecol_AcrR_MULT, Ecol_emrE,* and *AcrB,* which together form an efficient and regulated multidrug efflux pump system. Therefore, these nine transconjugants likely exhibit resistance to NAL as a result of the overexpression of this efflux pump. Further plasmid gene analysis revealed that approximately 25% of plasmids harbored dual resistance genes. Strikingly, 50% of the isolated conjugative plasmids derived from air samples carried ≥3 resistance genes, including the dominant air-borne plasmid peccDNA113 conferring resistance to 18 antibiotics – the highest number detected in this study. This demonstrates the extensive accumulation of multi-resistance genes within community environments, highlighting how the observed plasmid-mediated horizontal transfer poses a direct risk by rapidly converting susceptible bacteria into MDR strains. Critically, we detected the *blaNDM-5* gene, encoding the New Delhi metallo-β-lactamase (NDM) variant 5. This enzyme confers resistance to a broad spectrum of β-lactams and its dissemination across diverse replicon types likely contributes significantly to the observed high resistance rates in this environment (Wang-Kan et al., 2021; Poirel et al., 2010; Zong and Zhang, 2013). While a general concordance existed between phenotypic resistance and genetic determinants, certain exceptions were noted: transconjugants like WQ-5 exhibited resistance without detectable plasmid-borne genes, suggesting potential chromosomal or other vector-mediated resistance (Tyson et al., 2019). Conversely, isolates such as WK1/AK9 harbored the *Ecol_emrE* gene but lacked the corresponding phenotype, potentially due to low gene expression or resistance loss (Perlmutter et al., 2020), underscoring the complexity of resistance gene dissemination and expression. Analysis of virulence genes revealed that the dominant air-borne plasmid peccDNA113 carried the highest number of such genes. Its environmental dissemination could enhance pathogen fitness and create novel transmission routes (Hong et al., 2025; Agarwal et al., 2023). Of particular concern is its carriage of the *AcrB* gene, which combines multi-drug efflux capability with the ability to augment bacterial tissue invasiveness (Kobylka et al., 2020). Transfer of such a plasmid to pathogenic bacteria would dramatically increase therapeutic challenges and disease burden (Hu et al., 2024), blurring the lines between environmental and clinical resistance pools.

Furthermore, horizontal gene transfer (HGT) acts as the primary engine for cross-species ARG spread (Sezmis et al., 2023), with resistance fate determined by balancing fitness costs versus mobility. Environmental reservoirs like wastewater systems, particularly under anaerobic conditions, sustain resistant populations and facilitate HGT (Mantilla-Calderon and Hong, 2017), while persister cell subpopulations create resilient reservoirs that complete the transmission cycle between the environment and humans, a facet that warrants further investigation.

SNP-based phylogenetic analysis provided critical evidence for tracing plasmid dissemination pathways. Analysis of peccDNA113 showed that the air-derived plasmid AK59-2 and the wastewater-derived plasmid WK1-2 exhibited high homology with the Portugal mouse-associated plasmid CP054665.1, confirming its capacity for cross-species and cross-environmental transmission. Concurrently, air-derived plasmids AK56-2, AK9-2, and AK57-2 clustered closely with Sudan human tissue-associated plasmids CP092702.1/CP092699.1, supporting plasmid migration from human activity zones into the air medium. The prior detection of this plasmid lineage in US park lake water (2022) further corroborates its broad environmental adaptability. SNP analysis of plasmid *ps18D023_5* within wastewater sample WTOU33-5 revealed high homology with China wastewater-associated plasmids CP090570.1/CP090212.1, indicating the establishment of stable transmission chains within wastewater ecosystems. Furthermore, phylogenetic linkages connecting air and wastewater samples (e.g., AD73-2 and WK55-4) provide direct evidence for bidirectional resistance plasmid exchange between these environmental matrices. Crucially, this dispersal pattern closely mirrors that observed for clinically significant plasmids within hospital settings, underscoring the potential risk of plasmid flow across the community-healthcare interface. Collinearity analysis of the peccDNA113 plasmid identified a unique insertion sequence in the community-derived isolates compared to previously reported *peccDNA113* variants. This insert contained three functional gene modules: (1) The *dnaX* gene, acting as a replication enhancer by encoding the DNA polymerase III subunit tau, significantly boosts plasmid copy number (Zhang et al., 2018); (2) The *AcrB* gene, encoding the core component of the AcrAB-TolC multidrug efflux pump, confers resistance to macrolides, quinolones, β-lactams, and tetracyclines through active antibiotic extrusion (Wang et al., 2019; Kim et al., 2015); (3) An adaptation gene cluster (allB/fimC/fimH). The fimC/fimH operon enhances bacterial surface adhesion and colonization (Rodionova et al., 2023), while the presence of allB synergistically increases host niche competitiveness. Collectively, these findings indicate that the peccDNA113 plasmid identified within this residential community harbors an expanded repertoire of resistance and virulence genes compared to earlier environmental isolates, warranting significant concern.

A critical interpretation of our conjugation findings is necessary. The frequencies reported herein, while demonstrating transfer potential under controlled conditions, likely represent maximum theoretical values. Factors such as nutrient limitation and spatial structure in natural environments may significantly suppress conjugation efficiency (Piscon et al., 2023; Pillay et al., 2022). Moreover, reliance on antibiotic selection might miss transfers involving silent resistance genes. Therefore, these lab-derived values highlight the need for future studies to employ environmentally relevant conditions and metagenomic approaches for accurate risk assessment. This addresses the uncertainty regarding the representativeness of transfer rates under true environmental conditions.

Although the possibility of transformation or transduction cannot be completely ruled out, the standardized filter mating conditions used in this study are specifically designed to enrich for conjugation events. It is important to note that our study focused primarily on plasmid-mediated transfer. A key limitation is that our approach did not capture the contribution of other mobile genetic elements, such as ICEs, which are also major drivers of horizontal gene transfer in environmental bacteria. Furthermore, the use of *E. coli* as the sole recipient in our conjugation assay represents a methodological constraint. This approach is inherently biased toward capturing plasmids that are compatible with and can replicate in a Gram-negative host. Consequently, our study likely missed plasmids originating from Gram-positive bacterial donors, as these may not replicate in *E. coli* due to differences in replication machinery and host range.

Beyond conjugation efficiency, another critical factor influencing the long-term public health risk of these plasmids is their persistence and stability in bacterial populations within natural environments. Our study did not assess the fitness cost or long-term maintenance of these plasmids in the absence of antibiotic selection. Future investigations tracking the dynamics of these identified plasmids in complex microbial communities over time are essential to evaluate their resilience and potential for silent dissemination, which is critical to assessing their long-term public health impact.

While this study successfully captured transferable resistance plasmids and confirmed their conjugative potential from environmental samples, a methodological limitation exists in delineating the taxonomic origins (pathogenic vs. non-pathogenic bacteria) of the donor strains. However, the core threat lies in the plasmids’ intrinsic mobility: regardless of their original hosts, these mobile genetic elements can disseminate to clinically relevant pathogens, posing significant public health risks. Our approach deliberately prioritized characterizing environmentally disseminated plasmid vehicles over identifying transient donor reservoirs. This fundamentally differs from hospital-centric investigations focused on clinically derived resistance plasmids. By circumventing donor classification, we directly address a critical knowledge gap in AMR research: identifying the mobile resistome as the primary transmission engine, thereby shifting the focus toward intercepting resistance dissemination at its environmental source rather than at the point of human infection.

## 5 Conclusion

In summary, this study demonstrates the widespread presence of horizontally transferable antibiotic resistance plasmids in community air and wastewater environments. The majority of transconjugant isolates exhibited extensive resistance to ampicillin while maintaining susceptibility to polymyxin B. Particularly alarming was the detection of the carbapenemase gene *blaNDM-5*, which directly threatens last-resort antibiotic efficacy. Critically, we report the first discovery of the multidrug-resistant and virulence gene-carrying plasmid peccDNA113 in ambient air, whose environmental dissemination may accelerate resistant bacterial spread and synergistically exacerbate virulence risks. SNP analysis further confirmed bidirectional transmission of plasmids between wastewater and air, forming complex resistance gene networks. This highlights the pivotal role of airborne routes and environmental matrices in propagating resistance determinants, underscoring the urgent need for enhanced surveillance and targeted strategies to contain resistant strains and mitigate virulence threats.

## Data Availability

The original contributions presented in the study are publicly available. This data can be found in the NCBI GenBank database under the following accession numbers: PX560794, PX560795, PX560796, PX560797, PX560798, PX560799, PX560800, PX560801, PX560802, PX560803, PX651469, PX651722, PX560863, PX560864, PX560865, PX560866, PX560867, PX560917, PX560918, PX560919, PX560920, PX560921, PX560922, PX560923, PX560924, PX560925, PX560926, PX560932, PX560933, PX560934, PX560935, PX560936, PX244521, PX244522, PX132104, PX132105, PX560927, PX560928, PX677421, PX714844.

## Footnotes

1
https://ccbmicrobe.cs.uni-saarland.de/plsdb/

2
https://cloudtutu.com/
