# Supplementary material for: A hidden reservoir of antibiotic resistance genes: transferable plasmids in community air and wastewater
Source: Front Microbiol. 2026 Mar 5;17:1699056. doi: 10.3389/fmicb.2026.1699056 (PMC12999957; doi:10.3389/fmicb.2026.1699056)
Supplement: Supplementary file 1 [file Data_Sheet_1.docx]

**Appendix A. Supplementary material**

**Supplementary table 1.** Antibiotic susceptibility profiles of transconjugants

| **ID** | **Types of drug resistance** |
| --- | --- |
| CV601 | / |
| WK1 | AMP、CEP、TIG、NAL、CHL、AMK、TET |
| WK2 | CLR、MEM、CEP、NAL、CHL、AMK、TET |
| WK3 | CLR、AMP、MEM、CTX、ETP、SXT、CEP、CAZ、PB、GEN、CIP、TIG、NAL、STR、CHL、AMK |
| WK4 | CLR、AMP、ETP、SXT、CEP、CAZ、CIP、NAL |
| WQ5 | AMP、ETP、CEP、CAZ、GEN、CIP、NAL、AMK、TET |
| WQ6 | AMP、CEP、GEN、CIP、TIG、NAL、AMK、TET |
| AT7 | AMP、CEP、PB、CIP、TIG、NAL、AMK、TET |
| AT8 | AMP、SXT、CEP、CAZ、CIP、TIG、NAL、STR、CHL、AMK、TET |
| AK9 | AMP、CEP、TIG、NAL、CHL、AMK、TET |
| WT10 | CLR、AMP、MEM、CTX、ETP、SXT、CEP、CAZ、PB、GEN、CIP、TIG、NAL、STR、CHL、TET |
| AT11 | AMP、CTX、CEP、CIP、TIG、NAL、AMK、TET |
| AQ12 | AMP、CEP、CIP、NAL、AMK |
| WQ13 | AMP、ETP、CEP、NAL、AMK |
| WQ14 | AMP、CTX、ETP、SXT、CEP、CAZ、PB、CIP、TIG、NAL、STR、CHL、AMK、TET |
| WQ15 | AMP、SXT、CEP、GEN、TIG、NAL、STR、AMK、TET |
| WQ16 | AMP、SXT、GEN、NAL、STR、CHL、AMK、TET |
| WQ17 | CLR、AMP、CEP、GEN、CIP、TIG、NAL、STR、AMK、TET |
| AK18 | CLR、AMP、MEM、CEP、GEN、CIP、NAL、CHL、AMK、TET |
| BK19 | CLR、AMP、ETP、SXT、CEP、TIG、NAL、CHL、AMK、TET |
| WQ20 | AMP、CTX、CEP、GEN、CIP、NAL、AMK、TET |
| WQ21 | AMP、CEP、GEN、CIP、TIG、NAL、AMK、TET |
| WQ22 | CLR、AMP、MEM、CTX、ETP、SXT、CEP、CAZ、GEN、NAL、STR、AMK、TET |
| WQ23 | AMP、SXT、CIP、NAL、STR、CHL、AMK |
| AQ24 | AMP、CEP、GEN、CIP、NAL、AMK |
| AQ25 | AMP、CEP、PB、GEN、CIP、NAL、CHL、AMK |
| WQ26 | CLR、AMP、CTX、CEP、CAZ、GEN、TIG、NAL、STR、CHL、AMK、TET |
| WK27 | CLR、AMP、CTX、CEP、AMK、TET |
| WK28 | CLR、AMP、MEM、CEP、TIG、NAL、AMK、TET |
| WK29 | AMP、CEP、CIP、TIG、NAL、CHL、AMK、TET |
| WK30 | CLR、AMP、AMK、TET |
| WTOU31 | AMP、MEM、CTX、ETP、SXT、CEP、CAZ、GEN |
| WTOU32 | AMP、MEM、CTX、ETP、SXT、CEP、CAZ、NAL、STR、AMK |
| WTOU33 | AMP、MEM、CTX、ETP、SXT、CEP、CAZ、GEN、CIP、NAL、STR、CHL、TET |
| WTOU34 | AMP、MEM、CTX、ETP、SXT、CEP、CAZ、PB、NAL、STR、AMK |
| WTOU35 | AMP、MEM、CTX、ETP、SXT、CEP、CAZ、NAL、STR、AMK |
| WTOU36 | CLR、AMP、MEM、CTX、ETP、SXT、CEP、CAZ、PB、GEN、CIP、NAL、STR、AMK、TET |
| WS37 | AMP、MEM、CTX、ETP、CEP、AMK |
| WS38 | AMP、MEM、CTX、ETP、SXT、CEP、CAZ、GEN、TIG、NAL、STR、AMK、TET |
| WS39 | AMP、MEM、CTX、ETP、SXT、CEP、CAZ、CIP、TIG、NAL、STR、CHL、AMK、TET |
| WS40 | AMP、MEM、CTX、ETP、SXT、CEP、CAZ、GEN、NAL、STR、AMK |
| WTOU41 | AMP、MEM、CTX、ETP、SXT、CEP、CAZ、STR、AMK |
| WTOU42 | AMP、MEM、CTX、ETP、SXT、CEP、CAZ、NAL、STR、AMK、TET |
| WK43 | CLR、AMP、SXT、CEP、GEN、STR、CHL、AMK |
| WK44 | CLR、AMP、SXT、NAL、STR、CHL |
| WK45 | CLR、AMP、ETP、SXT、CEP、NAL、CHL、AMK、TET |
| WK46 | AMP、MEM、ETP、CEP、GEN、TIG、NAL、CHL、AMK、TET |
| AQ47 | AMP、CEP、GEN、CIP、NAL、AMK |
| AQ48 | AMP、CEP、CIP、NAL、CHL、AMK |
| WS49 | AMP、MEM、CTX、ETP、SXT、CEP、CAZ、TIG、NAL、STR、AMK |
| WS50 | AMP、MEM、CTX、ETP、SXT、CEP、CAZ、NAL、STR、AMK |
| WS51 | AMP、MEM、CTX、ETP、CEP、TIG、AMK、TET |
| WS52 | AMP、MEM、CTX、ETP、SXT、CEP、CAZ、NAL、STR、AMK、TET |
| WL53 | CLR、AMP、CTX、ETP、CEP、NAL、CHL、AMK、TET |
| WK54 | CLR、AMP、GEN、TIG、NAL、AMK、TET |
| WK55 | CLR、AMP、CEP、CIP、TIG、NAL、TET |
| AK56 | CLR、AMP、ETP、SXT、CEP、TIG、NAL、STR、CHL、AMK、TET |
| AK57 | AMP、CTX、SXT、CEP、CAZ、GEN、CIP、TIG、NAL、CHL、AMK、TET |
| WK58 | CLR、AMP、MEM、CTX、ETP、SXT、CEP、CAZ、STR、CHL、AMK、TET |
| AK59 | AMP、ETP、CEP、PB、TIG、NAL、CHL、TET |
| AD60 | ETP、CEP、TIG、NAL、CHL、AMK、TET |
| WD61 | AMP、CEP、TIG、NAL、AMK、TET |
| WS62 | AMP、MEM、CTX、ETP、CEP、CAZ、PB、NAL、AMK、TET |
| WS63 | AMP、MEM、ETP、CEP、AMK |
| WS64 | AMP、MEM、CTX、ETP、CEP、CAZ、TIG、NAL、AMK、TET |
| WS65 | AMP、MEM、ETP、CEP、CIP、AMK、TET |
| WS66 | AMP、CTX、ETP、CEP、CAZ、GEN、AMK、TET |
| WS67 | AMP、SXT、CEP、CHL |
| BD68 | AMP、ETP、CEP、PB、NAL、CHL、AMK、TET |
| BD69 | AMP、ETP、CEP、CAZ、TIG、NAL、CHL、AMK、TET |
| WD70 | AMP、CTX、CEP、GEN、NAL、CHL、AMK |
| WD71 | AMP、CEP、PB、NAL、CHL、AMK、TET |
| AD72 | AMP、SXT、CEP、CAZ、PB、CIP、NAL、STR、CHL、AMK、TET |
| AT73 | AMP、ETP、CEP、TIG、NAL、CHL、AMK、TET |
| AT74 | AMP、CEP、PB、TIG、NAL、CHL、AMK、TET |
| WS75 | AMP、MEM、CTX、ETP、CEP、CAZ、AMK |
| WS76 | AMP、CEP、PB、AMK |
| WS77 | AMP、MEM、ETP、CEP、CAZ、AMK、TET |
| WS78 | AMP、MEM、CTX、ETP、CEP、CAZ、AMK、TET |
| WQ79 | AMP、CEP、CIP、NAL、AMK、TET |
| WQ80 | AMP、CEP、GEN、CIP、AMK、TET |
| AQ81 | CLR、AMP、CEP、CIP、NAL、STR、AMK、TET |

**Supplementary table 2.** Plasmid Types, Suspected Origins, and Antibiotic Resistance Profiles

| ID | Plasmid | Type | Isolation source/Host | Source | Antibiotic | AGRs |
| --- | --- | --- | --- | --- | --- | --- |
| WQ16-2 | IncFIA/ IncFIB/ IncFIC | wastewater | chicken | *E. coli* | AMP、SXT、GEN、AMK、TET | *TEM-1、sul2、sul3、linG、aac(3)-IId、APH(3')-Ia、aadA17、tet(M) 、cmlA1、qacL、dfrA12、floR* |
| WTOU33-4 | IncFIA/ IncFIB/ IncFIC/ IncFII | wastewater | pig/Sus scrofa | *E. coli* | CAZ、ETP、SXT、AMP、CEP、NAL、CIP、GEN、STR、CHL、TET | *NDM-5, sul3, TEM-1, QnrS1, aac(3)-IId, aadA, cmlA1, aadA2, dfrA12, tet(A)* |
| WS39-2 | IncFIA/ IncFIB/ IncFIC/ IncX1 | wastewater | chicken | *E. coli* | AMP、MEM、CTX、SXT、CEP、 CAZ、TGC、AMK、TET | *aadA，cmlA1，aadA2，dfrA12，tet(A)，QnrS2，NDM-5，sul1，qacEdelta1，floR，sul2，tet(M)，APH(3')-Ia，aac(3)-IId，qacL* |
| WQ15-2 | IncFIA/ IncFIB/ IncU | wastewater | blood/human | *E. coli* | AMP、SXT、CEP、TGC、AMK、TET | *tet(A)，APH(6)-Id，APH(3'')-Ib，sul2，TEM-1，aac(3)-IId，dfrA17* |
| AQ12-2 | IncFIA/ IncFIC | air | human | *E. coli* |  |  |
| WQ17-2 | IncFIA/ IncFIC | wastewater | cucumber | *E. hormaechei* | CLR、AMP、CEP、GEN、CIP、TGC、 NAL、STR、AMK、TET | *APH(6)-Id，APH(3'')-Ib，sul2，mphA，Mrx，aac(3)-IId，QnrS1，TEM-1，tet(A)* |
| WTOU35-2 | IncFIA/ IncFIC | wastewater | human | *E. coli* | AMP、MEM、CTX、SXT、CEP、 CAZ、AMK | *qacEdelta1，aadA2，dfrA12，NDM-5，sul1* |
| WQ22-3 | IncFIA/ IncFII | wastewater | human | *E. coli* | AMP、CEP、CAZ、GEN、NAL、STR、AMK | *APH(3')-IIa，TEM-1，rmtB，CTX-M-55* |
| WQ26-2 | IncFIA/ IncFII/ IncQ1 | wastewater | pig/Sus scrofa | *E. coli* | CLR、AMP、CTX、CEP、CAZ、 GENSTR、CHL、AMK | *Erm(42)，sul2，Mrx，mphA，FosA3，aac(3)-IId，CTX-M-55，aadA23* |
| WK58-2 | IncFIB/ rep_cluster_2244 | wastewater | human | *E. coli* | CLR、AMP、SXT、CEP、STR、CHL、 AMK、TET | *tet(A)，APH(6)-Id，APH(3'')-Ib，sul2，TEM-1，mphA，Mrx，sul1，qacEdelta1，aadA5，dfrA17* |
| AT11-2 | IncFII/ IncN | air | urine/human | *K. pneumoniae* | CIP、TGC、NAL、TET | *QnrS1、tet(A)* |
| WK43-2 | IncI-gamma/K1 | wastewater | / | *S. enterica* | SXT、 STR | *Mrx，mphA，ErmB，sul2，APH(3'')-Ib，APH(6)-Id，dfrA17* |
| WQ23-3 | IncI-gamma/K1/ IncY | wastewater | trach wash | *E. coli* | AMP、SXT、CIP、NAL、STR、CHL、AMK | *aadA23，dfrA12，aadA2，sul3，aac(3)-IId，QnrS1，TEM-40* |
| WQ5-2 | IncN | wastewater | human | *S. flexneri* | AMP、CEP、CIP、NAL、AMK、TET | *TEM-1，tet(A)，QnrS1，aac(3)-Iic* |
| WQ14-2 | IncN | wastewater | / | *S. enterica* | AMP、CAZ、CEP、TET | *TEM-1, rmtB* |
| WK55-2 | IncN | wastewater | / | *E. coli* | CLR、CEP、TGC、TET | *MphA, Mrx, tet(A)* |
| AD72-4 | IncW | air | / | *E. hormaechei* | SXT、AMK | *sul1，qacEdelta1，aadA2，aac(6')-Ib9* |
| WK1-2 | peccDNA113 | wastewater | transconjugant/lake | *E. coli* | AMP、CEP、TGC、NAL、CHL、AMK、TET | *Ecol_emrE，AcrB，Ecol_AcrA，Ecol_AcrR_MULT* |
| AK9-2 | peccDNA113 | air | transconjugant/lake | *E. coli* | AMP、CEP、TGC、NAL、CHL、AMK、TET | *Ecol_emrE，AcrB，Ecol_AcrA，Ecol_AcrR_MULT* |
| AK18-2 | peccDNA113 | air | transconjugant/lake | *E. coli* | CLR、AMP、MEM、CEP、GEN、CIP、 NAL、CHL、AMK、TET | *Ecol_emrE，Ecol_AcrR_MULT，Ecol_AcrA，AcrB* |
| AK56-2 | peccDNA113 | air | transconjugant/lake | *E. coli* | CLR、AMP、SXT、CEP、TGC、NAL、 STR、CHL、AMK、TET | *Ecol_emrE，Ecol_AcrR_MULT，Ecol_AcrA，AcrB* |
| AK57-2 | peccDNA113 | air | transconjugant/lake | *E. coli* | AMP、CTX、SXT、CEP、CAZ、GEN、 CIP、TGC、NAL、CHL、AMK、TET | *Ecol_emrE，AcrB，Ecol_AcrA，Ecol_AcrR_MULT* |
| AK59-2 | peccDNA113 | air | transconjugant/lake | *E. coli* | AMP、CEP、PB、TGC、NAL、CHL、TET | *Ecol_emrE，AcrB，Ecol_AcrA，Ecol_AcrR_MULT* |
| AT11-3 | ps15D023_8 | air | transconjugant/lake | *S. enterica* | TGC、TET | *Ecol_EFTu_PLV* |
| WQ22-4 | ps15D023_8 | wastewater | human | *S. enterica* | STR、AMK、TET | *Ecol_EFTu_PLV* |
| WTOU33-5 | ps15D023_8 | wastewater | human | *S. enterica* | TET | *Ecol_EFTu_PLV* |
| WK43-4 | ps15D023_8 | wastewater | human | *S. enterica* | SXT | *Ecol_EFTu_PLV* |
| WK55-4 | ps15D023_8 | wastewater | human | *S. enterica* | TGC、TET | *Ecol_EFTu_PLV* |
| WK58-3 | ps15D023_8 | wastewater | human | *S. enterica* | TET | *Ecol_EFTu_PLV* |
| AD60-2 | ps15D023_8 | air | human | *S. enterica* | TGC、TET | *Ecol_EFTu_PLV* |
| AT73-2 | ps15D023_8 | air | human | *S. enterica* | TGC、TET | *Ecol_EFTu_PLV* |
| AQ12-3 | rep_cluster_1760 | air | / | *E. coli* | / | */* |
| WQ23-5 | rep_cluster_1760 | wastewater | / | *E. coli* | / | */* |
| AQ47-2 | rep_cluster_1760 | air | / | *E. coli* | CIP、NAL、AMK | *QnrS1、aac(3)-IId* |
| AD72-3 | rep_cluster_1760 | air | / | *E. coli* | / | */* |
| WK55-3 | rep_cluster_312 | wastewater | pig/Sus scrofa | *E. coli* | / | */* |
| WQ23-4 | rep_cluster_488 | wastewater | pig/Sus scrofa | *E. coli* | / | */* |
| WQ17-3 | rep_cluster_867 | wastewater | Human feces/human | *E. coli* | / | / |
| WTOU33-3 | unnamed15 | wastewater | human | *K. oxytoca* | AMP、MEM、CTX、SXT、CEP、CAZ、GEN、CIP、NAL、STR、CHL、TET | *mdtE、mdtF、gadX、cpxA* |
| WS39-3 | unnamed15 | wastewater | human | *K. oxytoca* | AMK、GEN | *cpxA* |

**Supplementary table 3.** Distribution of virulence genes of plasmids

| Name | Number | Rate (%) | Possible Source |
| --- | --- | --- | --- |
| *fimD* | 7 | 17.50% | *C. freundii* |
| *hemE* | 7 | 17.50% | *H.somnus* |
| *AcrB* | 7 | 17.50% | *K.pneumoniae* |
| *fimH* | 7 | 17.50% | *S.enterica* |
| *entS* | 6 | 15.00% | *E.coli* |
| *fepB* | 6 | 15.00% | *E.coli* |
| *fepE* | 6 | 15.00% | *E.coli* |
| *fes* | 6 | 15.00% | *E.coli* |
| *ibeB* | 6 | 15.00% | *E.coli* |
| *KOX_12990* | 6 | 15.00% | *K.oxytoca* |
| *allA* | 6 | 15.00% | *K.pneumoniae* |
| *allB* | 6 | 15.00% | *K.pneumoniae* |
| *allC* | 6 | 15.00% | *K.pneumoniae* |
| *allD* | 6 | 15.00% | *K.pneumoniae* |
| *allR* | 6 | 15.00% | *K.pneumoniae* |
| *allS* | 6 | 15.00% | *K.pneumoniae* |
| *fepG* | 6 | 15.00% | *S.boydii* |
| *entC* | 6 | 15.00% | *S.dysenteriae* |
| *entA* | 6 | 15.00% | *S.dysenteriae* |
| *entE* | 6 | 15.00% | *S.dysenteriae* |
| *fepC* | 6 | 15.00% | *S.dysenteriae* |
| *ompD* | 6 | 15.00% | *S.enterica* |
| *entB* | 6 | 15.00% | *S.flexneri* |
| *entD* | 6 | 15.00% | *S.flexneri* |
| *fepA* | 6 | 15.00% | *S.flexneri* |
| *fepD* | 6 | 15.00% | *S.flexneri* |
| *entF* | 6 | 15.00% | *S.sonnei* |
| *traJ* | 6 | 15.00% | *E.coli* |
| *sitA* | 3 | 7.50% | *E.coli* |
| *sitB* | 3 | 7.50% | *E.coli* |
| *sitC* | 3 | 7.50% | *E.coli* |
| *sitD* | 3 | 7.50% | *E.coli* |
| *iucA* | 2 | 5.00% | *E.coli* |
| *iucB* | 2 | 5.00% | *E.coli* |
| *iucC* | 2 | 5.00% | *E.coli* |
| *iucD* | 2 | 5.00% | *E.coli* |
| *iutA* | 2 | 5.00% | *E.coli* |
| *Fphi_1039* | 2 | 5.00% | *F.philomiragia* |
| *others* | 36 | 90.00% | */* |

**Supplementary table 4.** Summary of GenBank Accession Numbers, Sequences and Isolation Sources

| **GenBank Accession Number** | **Sequence Name** | **Isolation Source** |
| --- | --- | --- |
| CP054665.1 | *Ecoil IGC_EcoliInv_1.1* | Mus musculus (G2 mouse) |
| NZ_CP110860.1 | *peccDNA113* | TSW4848-W1 1 is a transconjugant of SW4848 |
| CP092702.1 | *Ecoil S-P-C-029.01* | human |
| CP092699.1 | *Ecoil S-P-N-063.01* | human |
| CP101347.1 | *ps15D023_8* | human |
| CP090570.1 | *Ecoil BY63* | wastewater |
| CP090212.1 | *Ecoil BY68* | wastewater |
| CP037857.2 | *Ecoil BW25113* | human |
| CP139657.1 | *Ecoil TP10-3* | hospital |
| CP139677.1 | *Ecoil TP6-1* | hospital |
| CP139673_1 | *Ecoil TP6-2* | hospital |
| CP139669_1 | *Ecoil TP6-3* | hospital |
| CP139665_1 | *Ecoil TP10-1* | hospital |
| CP139661_1 | *Ecoil TP10-2* | hospital |

**Supplementary table 5.**  GenBank Accession Numbers of Plasmids

| ID | GenBank Accession Numbr |
| --- | --- |
| WQ14-2 | PX560794 |
| WQ15-2 | PX560795 |
| WQ16-2 | PX560796 |
| WQ17-2 | PX560797 |
| WQ17-3 | PX560798 |
| AT11-2 | PX560799 |
| AT11-3 | PX560800 |
| AQ12-2 | PX560801 |
| AQ12-3 | PX560802 |
| WQ5-2 | PX560803 |
| WTOU33-4 | PX651469 |
| WTOU35-2 | PX651722 |
| WS39-2 | PX560863 |
| WS39-3 | PX560864 |
| WK43-2 | PX560865 |
| WK43-4 | PX560866 |
| AQ47-2 | PX560867 |
| WK58-2 | PX560917 |
| WK58-3 | PX560918 |
| AK59-2 | PX560919 |
| AD60-2 | PX560920 |
| AD72-3 | PX560921 |
| WQ22-3 | PX560922 |
| WQ22-4 | PX560923 |
| WQ23-3 | PX560924 |
| WQ23-4 | PX560925 |
| WQ23-5 | PX560926 |
| WK55-2 | PX560932 |
| WK55-3 | PX560933 |
| WK55-4 | PX560934 |
| AK56-2 | PX560935 |
| AK57-2 | PX560936 |
| WK1-2 | PX244521 |
| AK18-2 | PX244522 |
| WTOU33-5 | PX132104 |
| AK9-2 | PX132105 |
| AD72-4 | PX560927 |
| AT73-2 | PX560928 |
| WTOU33-3 | PX677421 |
| WQ26-2 | PX714844 |
