# Supplementary figures and images for: A hidden reservoir of antibiotic resistance genes: transferable plasmids in community air and wastewater
Source: Front Microbiol. 2026 Mar 5;17:1699056. doi: 10.3389/fmicb.2026.1699056 (PMC12999957; doi:10.3389/fmicb.2026.1699056)

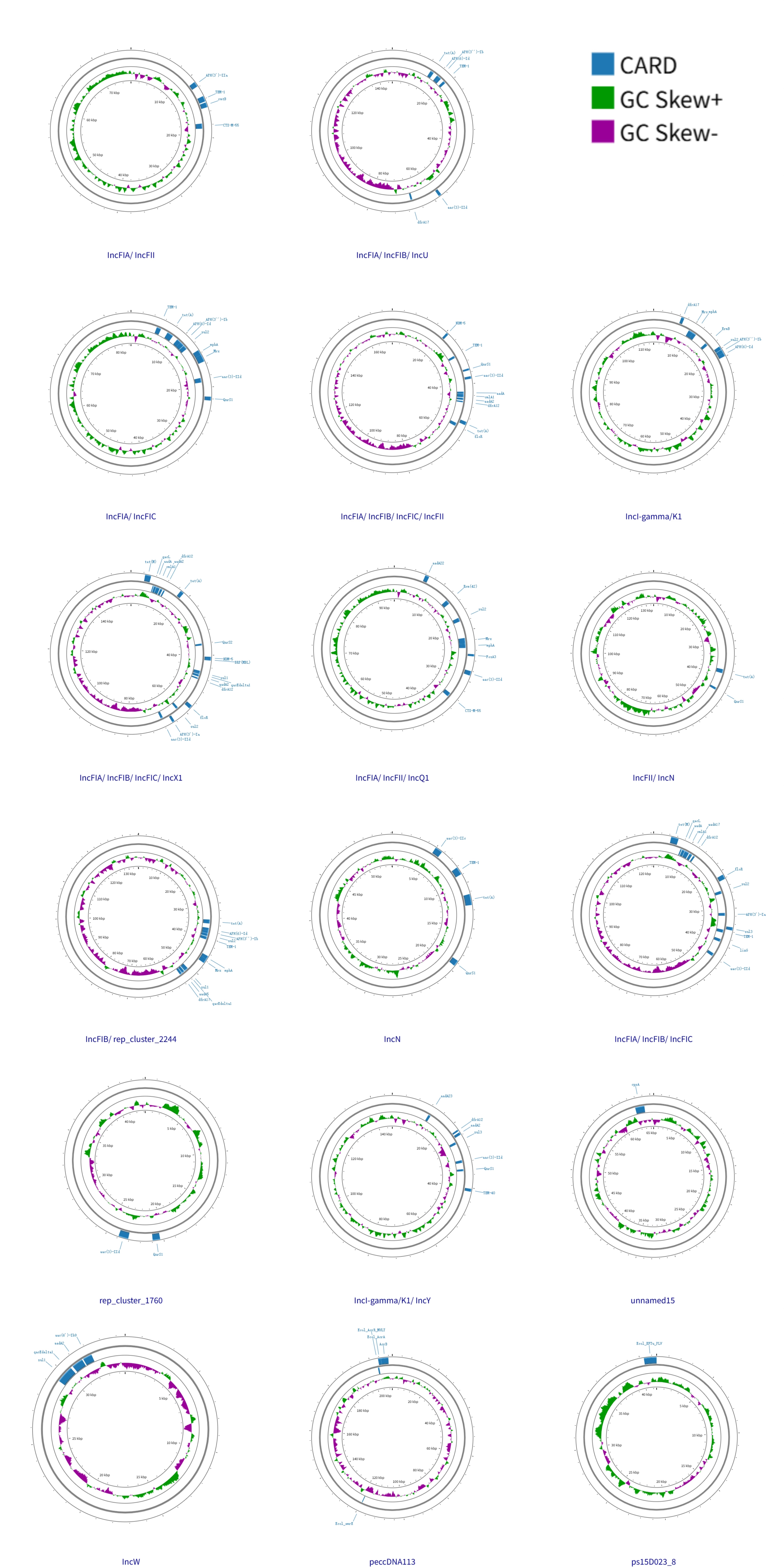

Supplement: Supplementary file 2 [file Image_1.jpeg]
